# Mean diffusivity related to collectivism among university students in Japan

**DOI:** 10.1038/s41598-018-37995-5

**Published:** 2019-02-04

**Authors:** Seishu Nakagawa, Hikaru Takeuchi, Yasuyuki Taki, Rui Nouchi, Yuka Kotozaki, Takamitsu Shinada, Tsukasa Maruyama, Atsushi Sekiguchi, Kunio Iizuka, Ryoichi Yokoyama, Yuki Yamamoto, Sugiko Hanawa, Tsuyoshi Araki, Carlos Makoto Miyauchi, Daniele Magistro, Kohei Sakaki, Hyeonjeong Jeong, Yukako Sasaki, Ryuta Kawashima

**Affiliations:** 1 Division of Psychiatry, Tohoku Medical and Pharmaceutical University, Sendai, Japan; 2 Department of Human Brain Science, Institute of Development, Ageing and Cancer, Tohoku University, Sendai, Japan; 3 Division of Developmental Cognitive Neuroscience, Institute of Development, Ageing and Cancer, Tohoku University, Sendai, Japan; 4 Division of Medical Neuroimaging Analysis, Department of Community Medical Supports, Tohoku Medical Megabank Organization, Tohoku University, Sendai, Japan; 5 Department of Nuclear Medicine and Radiology, Institute of Development, Ageing and Cancer, Tohoku University, Sendai, Japan; 6 Creative Interdisciplinary Research Division, Frontier Research Institute for Interdisciplinary Science (FRIS), Tohoku University, Sendai, Japan; 7 Smart Ageing International Research Center, Institute of Development, Ageing and Cancer, Tohoku University, Sendai, Japan; 8 Department of Behavioral Medicine, National Institute of Mental Health, National Center of Neurology and Psychiatry, Kodaira, Tokyo, Japan; 9 Department of Psychiatry, Tohoku University Graduate School of Medicine, Sendai, Japan; 10 School of Medicine, Kobe University, Kobe, Japan; 11 ADVANTAGE Risk Management Co., Ltd, Tokyo, Japan; 12 Department of Language Sciences, Graduate School of Humanities, Tokyo Metropolitan University, Tokyo, Japan; 13 Department of Sport Science, School of Science and Technology, Nottingham Trent University, Nottingham, UK; 14 Advanced Brain Science, Institute of Development, Aging and Cancer, Tohoku University, Sendai, Japan; 15 Graduate School of International Cultural Studies, Tohoku University, Sendai, Japan

## Abstract

Collectivism is an important factor for coping with stress in one’s social life. To date, no imaging studies have revealed a direct association between collectivism and white matter structure. Collectivism is positively related to independence, harm avoidance, rejection sensitivity, cooperativeness, external locus of control, and self-monitoring and negatively related to need for uniqueness. Accordingly, we hypothesised that the neural structures underpinning collectivism are those that are also involved with its relationship using magnetic resonance imaging (MRI). This study aimed to identify the brain structures associated with collectivism in healthy young adults (n = 797), using regional grey and white matter volume, fractional anisotropy, and mean diffusivity (MD) analyses of MRI data. Scores on the collectivism scale were positively associated with MD values in the bilateral dorsolateral prefrontal cortex, left orbitofrontal cortex, inferior frontal gyrus, right superior temporal gyrus, ventral posterior cingulate cortex, globus pallidus, and calcarine cortex using the threshold-free cluster enhancement method with family-wise errors corrected to *P* < 0.05 at the whole-brain level. No significant associations between were found collectivism and other measures. Thus, the present findings supported our hypothesis that the neural correlates of collectivism are situated in regions involved in its related factors.

## Introduction

Collective coping behaviours among culturally diverse populations are important for stress coping processes via collectivistic values^1^. Enhancing prosocial behaviour in collectivism is an effective means of social scrutiny^2^. Collectivism is defined as a social pattern that links individuals to parts of collectives (family, tribe, nation, etc.)^3^. Personal collectivism is defined as the tendency of an individual to prioritise the collective self over the private self, when those are in conflict^4^. Collectivism scale scores have been positively related to interdependence (highly correlated concept of collectivism), rejection from the group (related to harm avoidance), self-monitoring, and being an external locus of control, whereas they are negatively correlated with need for uniqueness^4^. Furthermore, collectivism increases cooperativeness^5^, particularly in Japanese participants^6^.

Collectivism has also been studied using functional magnetic resonance imaging (fMRI). Temporarily heightened awareness of individualist or collectivist orientations is sufficient to elicit greater activation within the medial prefrontal cortex (mPFC) and posterior cingulate cortex (PCC), respectively, during general or contextual self-descriptions^7^. Moreover, the degree of agreement with individualistic or collectivistic values, respectively, predicted mPFC responses to general or contextual self-descriptions^8^. Collectivists showed stronger activation in the left temporoparietal regions than individualists, who mainly recruited the medial prefrontal regions^9^. Importantly, fMRI is based in hemodynamics and is only an indirect measure of brain activation^10^. Excitatory brain activity remains unable to be distinguished from inhibitory actions by fMRI^11^. In brief, we could just say that ‘this area is associated with processing stimuli’^11^.

Numerous structural studies of the brain have assessed the neural correlates of factors related to collectivism. First, with respect to factors positively related to collectivism, interdependence using questionnaires was associated with decreased grey matter volume (GMV) limited in PFC (ventromedial PFC [vmPFC], dorsolateral PFC [DLPFC], rostrolateral PFC [RLPFC]^12^, and orbitofrontal cortex [OFC])^13^. Harm avoidance identified by the use of questionnaires was positively correlated with mean diffusivity (MD) in the globus pallidus^14^, putamen^15^, and superior temporal gyrus (STG)^14^. Cooperativeness, also assessed by questionnaire, was negatively correlated with fibre connectivity for the cognitive control system from the dorsal caudate to the rostral cingulate cortex and ventrolateral PFC^16^. Self-monitoring is inversely associated with white matter (WM) integrity in the anterior cingulum, which connects the dorsal anterior cingulate cortex and midline structures^17^. Furthermore, white matter volume (WMV) in the striatum showed significant negative correlations with a sense of being the external locus of control^18^. Second, with respect to factors negatively related to collectivism, individual need for uniqueness was also associated with larger WMV^19^ of the body of the corpus callosum.

Although MD and fractional anisotropy (FA) measure are related parameters of diffusion tensor imaging (DTI), they were different microstructural brain properties. Affection of MD includes capillaries, spines, and macromolecular proteins, properties of myelin, membrane, axon, the shape of neurons, protoplasmic glia, fibrous glia, and enhanced tissue organisation^20^. On the other hand, FA is more strongly associated with microstructural properties related to brain connectivity and sensitive to increases in axonal membrane thickness, diameter, and/or the amount of parallel organisation of the axons^20^. The general trend during youth is increasing FA and decreasing mean MD with age^21^. Accordingly, MD and FA may be used together to supplement the strengths of each method.

Previous studies have proved that neural correlates of collectivism were only the PFC, especially mPFC by fMRI studies^7,8^ and grey matter structure studies^12,13^, although collectivism could be related to several aforementioned psychological factors. Accordingly, the present study hypothesised that the degree of collectivism is related to the neural correlates of factors related to collectivism (the PFC, temporoparietal junction [TPJ], PCC, and striatum), and that this relationship can be demonstrated using regional WMV (rWMV), FA, and MD analyses. We also checked correlation between collectivism scores and regional GMV (rGMV) for reproducibility of the outcomes of the previous findings (i.e., the PFC)^12,13^. Thus, the present study aimed to identify the neuroanatomical correlates of collectivism in young people.

## Results

### Behavioral Data

**Figure 1:**
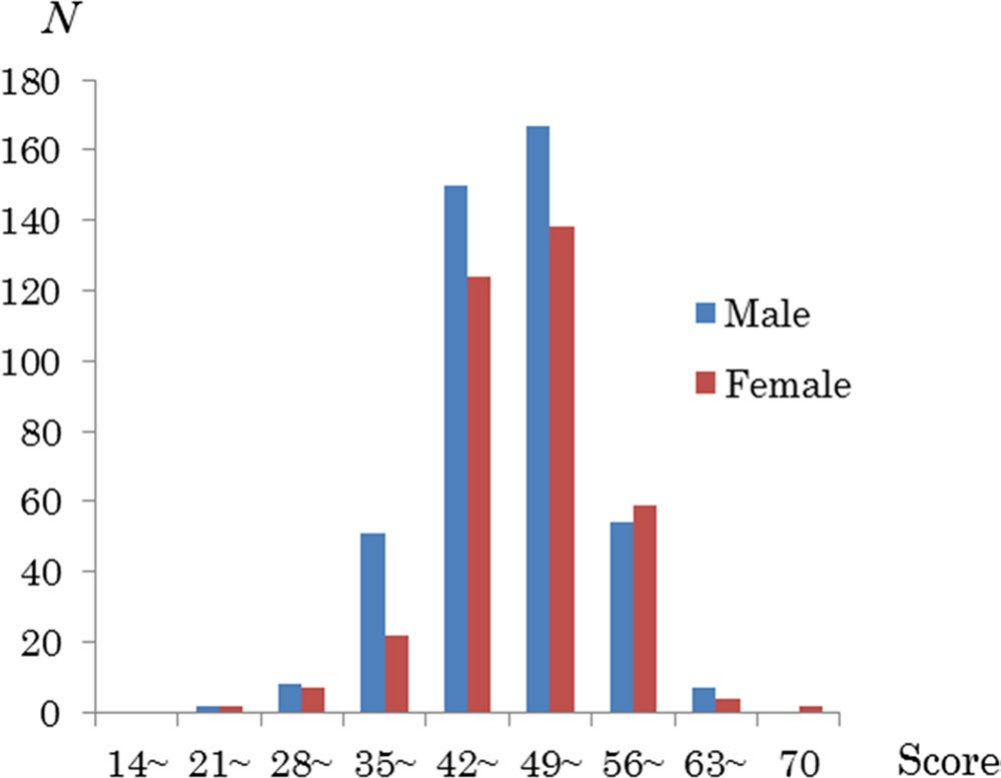
Distribution of collectivism scores in males and females. Histograms showing the distribution of collectivism scores in men and women.

Table 1 shows the means and standard deviations (SD) for age, and scores for the Raven’s Advanced Progressive Matrix (RAPM) and collectivism scale. Figure 1 depicts the distributions of the collectivism scores among men and women.

**Table 1:** Sex differences in age and scores (mean ± SD) on the RAPM, and collectivism; and one-way ANOVA results.

Measure	Total (SD)	Males (*N* = 439)	Females (*N* = 358)	*P*	*F*
Age	20.6 (1.8)	20.7 (1.9)	20.5 (1.6)	0.037*	4.4
RAPM	28.5 (3.8)	28.9 (3.7)	28.0 (3.9)	0.002**	10.1
Collectivism	43.0 (6.7)	42.7 (6.7)	43.3 (6.8)	0.171	1.9

*P < 0.05, **P < 0.01.

ANOVA, analysis of variance; RAPM, Raven’s Advanced Progressive Matrix; SD, standard deviation.

After adjustment for the effects of age, sex, and RAPM, significant correlation among the collectivism scores was observed among the 797 subjects (*P* < 0.001).

There was significant difference between men and women in the RAPM scores (*P* < 0.05, one-way analysis of variance [ANOVA]) but not the collectivism scores (*P* = 0.171).

### Magnetic resonance imaging Data

#### Analysis of voxel-based morphometry (VBM) data

After adjustment for sex, age, total intracranial brain volume (TIV; total GM volume + total WM volume + total cerebrospinal fluid [CSF] volume), and RAPM scores, there were no significant positive or negative correlations between collectivism scores and rGMV (or rWMV) at each voxel at a family-wise errors (FWE)-corrected threshold of *P* < 0.05 based on the threshold-free cluster enhancement (TFCE) method at the whole-brain level. There were negative correlations between collectivism scores and rGMV (or rWMV) at each voxel at a threshold of *P* < 0.001 uncorrected (*t* = 3.5) at the whole-brain level. For detailed results, please see Supplemental Table 1.

#### Regions of interest (ROI) analysis of the association between collectivism and rGMV

After adjustment for the aforementioned confounding factors, there were no significant positive or negative correlations between collectivism scores and rGMV in ROIs at the aforementioned threshold.

#### Analysis of MD and FA data

**Figure 2:**
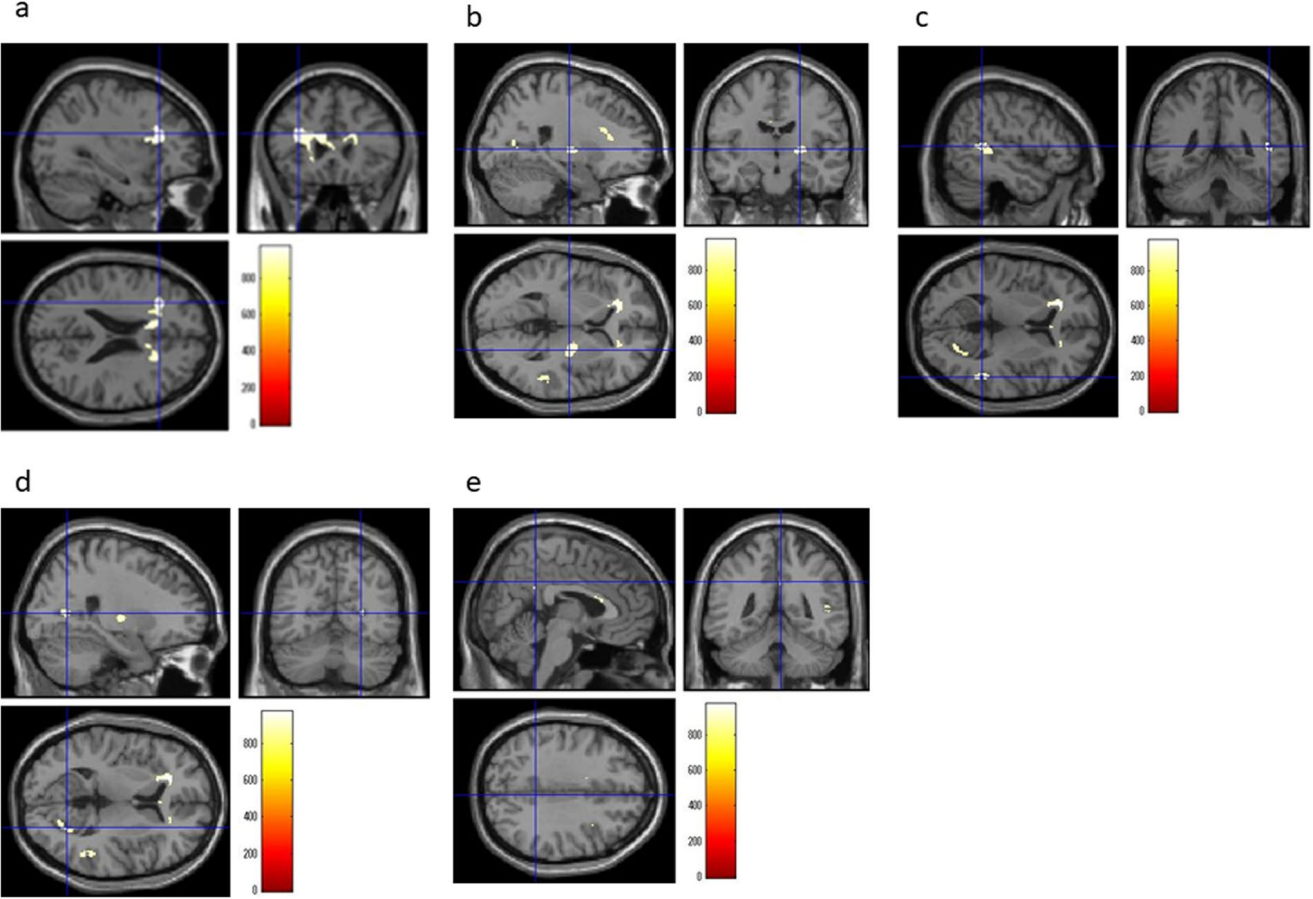
Regions correlated with MD and collectivism scores. The present results were determined based on an FWE-corrected threshold of *P* < 0.05 with a TFCE based on 5000 permutations; the results were corrected at the whole-brain level. Regions showing correlations were overlaid on a single T1 image in the SPM8 toolbox. The red-to-yellow colour scale indicates the strength of the TFCE value for the positive correlation between the MD and collectivism scores; areas with significant correlations were identified in the left DLPFC (**a**), the right globus pallidus (**b**), STG (**c**), calcarine (**d**), and vPCC (**e**). DLPFC: dorsolateral prefrontal cortex, FWE: family-wise errors, vPCC: ventral posterior cingulate cortex, MD: Mean diffusivity, STG: Superior temporal gyrus, TFCE: threshold-free cluster enhancement.

**Figure 3:**
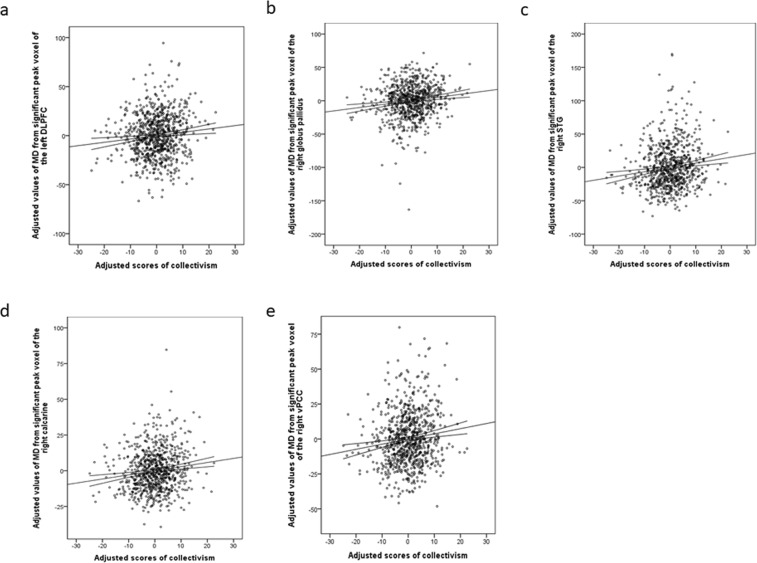
Peak regional MD values of the significant regions and scores of collectivism. Residual plots with trend lines depicting the correlations between residuals in the multiple regression analysis with collectivism scores as the dependent variable and other confounding factors as independent variables; 95% confidence intervals for the trend lines are shown. The peak regional MD values of the significant regions in the left DLPFC (**a**), the right globus pallidus (**b**), STG (**c**), calcarine cortex (**d**), and vPCC (**e**). DLPFC: dorsolateral prefrontal cortex, vPCC: ventral posterior cingulate cortex, MD: Mean diffusivity, STG: Superior temporal gyrus.

A whole-brain multiple regression analysis after adjustment for sex, age, TIV, and RAPM scores, revealed a significant positive correlation between collectivism scores and MD in the bilateral DLPFC, left OFC, inferior frontal gyrus (IFG), right globus pallidus, STG along with the posterior superior temporal sulcus, calcarine cortex, and ventral PCC (vPCC), using the TFCE method with FWE corrected to *P* < 0.05 at the whole-brain level (Table 2; Figs 2 and 3). There were no significant negative correlations between collectivism scores and MD at the same threshold at the whole-brain level. Most peak voxels of the significant regions except for one peak voxel of vPCC and that of the globus pallidus fall in white matter.

**Table 2:** Brain regions exhibiting a significant correlation between MD and collectivism scores.

Brain region	G/W	R/L	x	y	z	*TFCE* *Value*	Corrected *P-*value (FWE)	Cluster size (k_E_)	*β*
DLPFC	W	L	−32	26	23	971.7	0.034*	2309	0.129
OFC	W	L	−17	35	−6	971.1	0.034*		
IFG	W	L	−21	33	8	963.3	0.034*		
DLPFC	W	R	36	11	38	868.7	0.046*	24	0.145
Globus pallidus	G	R	24	−14	3	909.2	0.040*	175	0.142
STG	W	R	51	−44	8	906.7	0.040*	160	0.136
MTG	W	R	53	−36	3	895.4	0.041		
MTG	W	R	56	−35	3	847.9	0.050*	1	0.117
Calcarine cortex	W	R	26	−63	8	886.7	0.043*	84	0.144
vPCC	W	R	5	−45	35	849.2	0.050*	3	0.136
vPCC	G	R	5	−47	29	846.6	0.050*	2	0.130

*P < 0.05, FWE corrected.

DLPFC, dorsolateral prefrontal cortex; FWE, family-wise errors; G, grey matter; IFG, inferior frontal gyrus; L, left; R, right; MD, Mean diffusivity; OFC, orbitofrontal cortex; STG, Superior temporal gyrus; TFCE, threshold-free cluster enhancement; vPCC, ventral posterior cingulate cortex; W, white matter.

We found no significant correlations between collectivism scores and FA using the same analyses mentioned above. Further, there was no correlations between collectivism scores and FA at each voxel at a threshold of *P* < 0.001 uncorrected (*t* = 3.5) at the whole-brain level.

#### Interaction effects of sex and collectivism on brain structures

Using data from both sexes with respect to the covariates of age, RAPM, TIV, and collectivism scores, an analysis of covariance (ANCOVA) revealed no significant interaction effect between collectivism scores and sex on rGMV, rWMV, FA, or MD using the TFCE method with FWE corrected to *P* < 0.05 at the whole-brain level.

## Discussion

To our knowledge, the present study is the first to investigate the direct associations between collectivism and white matter structures in healthy individuals at the whole-brain level. Consistent with the stated hypothesis, total collectivism scores were most positively associated with MD values in the left PFC (including DLPFC, OFC, and IFG), the right DLPFC, STG (including TPJ), vPCC, and globus pallidus. These brain structural outcomes confirmed that the regions implicated in functional studies of collectivism are also associated with the neural correlates of interdependence, rejection sensitivity, cooperativeness, self-monitoring, and external locus of control.

There are several mechanisms potentially underlying the significant relationship between collectivism and the regions implicated here: the PFC (the bilateral DLPFC, left orbitofrontal cortex, and IFG) in accordance with the previous grey matter structural studies associated with independence^12,13^. Furthermore, cooperativeness is positively related to MD in the PFC^14^, whereas self-monitoring is inversely associated with WM integrity in the anterior cingulum^17^. Thus, low function of the PFC including anterior cingulum could be main neural correlates of collectivism as aspects of interdependence, cooperativeness, and self-monitoring.

We also demonstrated that collectivism scores were related to higher MD values in the globus pallidus and the vPCC. Harm avoidance (positively related to collectivism) was positively correlated with MD in the basal ganglia^14^. Moreover, the external locus of control (positively related to collectivism) was negatively correlated with WMV in the striatum^18^. With respect to the vPCC, GMV in the region of the PCC has been negatively associated with rejection sensitivity^22^. Uniquely, one of the peak voxels of the significant regions at vPCC falls in the grey matter. Increasing MD, especially in grey matter includes reduction of spines and macromolecular proteins, protoplasmic glia^20^. Accordingly, the low function of the globus pallidus and vPCC seemed to be neural correlates of collectivism as aspects of harm avoidance, rejection sensitivity, and external locus of control.

The calcarine cortex was unexpectedly related to collectivism. Collectivism scale scores were positively related to social anxiety^4^. Interestingly, compared with healthy controls, decreased functional connectivity between the frontal and occipital lobes might be related to abnormal information processing in patients with social anxiety disorder in resting-state fMRI^23^. Furthermore, lower nodal centralities in patients with social anxiety disorder were seen in the calcarine, DLPFC, and insula than those of healthy controls in resting-state fMRI^24^. Accordingly, based on the social anxiety, the dysfunction of the calcarine cortex is therefore presumed to be a legitimate neural correlate of collectivism. Notably, all MD values in regions significantly related to collectivism were not negative but positive relationships. MD decreases are extraordinarily sensitive to white matter development, reflecting underlying changes in tissue water content and cytoarchitecture^25^. Nonetheless, the lower tissue density in the regions was related to the tendency for collectivism. This interpretation is in accordance with the idea that collectivists would perceive disruption to their interdependence, social harmony, and security as more threatening and stressful than individualists, as recently suggested in a review of the subject^1^.

Moreover, there were no significant associations between collectivism and other measures (rGMV, rWMV, and FA) at the standard threshold. A plausible reason for this is that proliferation of glial cells, such as oligodendrocytes, astrocytes, and microglia might be particularly affected by decreasing MD in white matter. These cells substantially impact neuronal function and activities^26^, for example, by supporting axons by regulating the extracellular ion concentration^27^. In addition, DTI results particularly indicate white matter maturation during adolescence^28^. Young adults in this study might show higher collectivism scores due to developmentally slower maturation. Most MD regions (i.e., frontal regions and STG [including TPJ]) significantly associated with collectivism seem to develop even after adolescence. Notably, the frontal-temporal connections have the most prolonged development^29^. Furthermore, FA values were reduced 3–11% after the peak (20–42 years old), whereas MD values increased 4–20% after the minimum (18–43 years old)^29^. This difference in the magnitude of changing values might affect the sensitivity to detect regions associated with collectivism. It has been postulated that people typically become more collectivist with age, with one of the main reasons being that older people might have more established social relationships than younger people^3^. With respect to differences among similar studies, the mean age of participants of the study by Kitayama *et al*.^13^ was 26.4 (range 20–39) years, which was older than that of the participants in our study. The finding by Wang *et al*.^12^ does not seem to survive at our stringent statistical threshold; their statistical threshold was uncorrected *P* < 0.005 at whole brain level. Accordingly, only MD could be significantly associated with the values of collectivism in our study.

Finally, as we have previously noted^30,31^, there are a few limitations of this study. Because the present study used a cross-sectional design, the results cannot be used to determine the causality between collectivism and brain regions with which it is significantly associated. Thus, to overcome this limitation, a prospective study that confirms such causality is necessary. Furthermore, we used young healthy subjects who possessed high levels of education, which may affect the ability of our results to be generalised to other groups. Education tends to individualism due to exposure to cultural diversity^3^. Accordingly, future research is necessary to study neural correlates of collectivism with subjects differing in culture, education, and generation.

## Methods

### Subjects

The present study included 797 healthy right-handed individuals (439 men and 358 women; mean age: 20.6 ± 1.8 years). Written informed consent was obtained from each participant prior to the study in accordance with the Declaration of Helsinki (1991). All study procedures were approved by the Ethics Committee of Tohoku University, and all experiments were performed in accordance with the approved guidelines. For more details regarding the study procedures, please see our previous studies^32–34^.

### Psychological Outcome Measures

#### Assessment of collectivism

We used Yamaguchi’s (revised) Collectivism Scale to measure the allocentric tendency to prioritise a group goal over personal goal when the two goals are in conflict. The scale was developed by adding four individualist items to an original scale^4^ for application to both collectivists and individualists^35^: for example, ‘I stick with my group even through difficulties’, ‘I maintain harmony in my group’, and ‘I respect decisions made by my group’. The validity of the revised scale for the variables has been demonstrated to be consistent across cultural groups because the total collectivism scores are associated with higher affiliative tendency, higher rejection sensitivity, and lower need for uniqueness in three countries (the United States, Korea and Japan)^35^. Thus, this psychometric scale appears to represent a cognitive component present across groups and yields meaningful relationships with other psychological constructs. The total score of the revised scale has satisfactory internal consistency for all countries^35^. This scale includes a 14-item questionnaire, each item rated on a 5-point Likert scale from *describes me not at all* (1) to *describes me very well* (5), which results in a total score ranging from 14 to 70, where higher scores indicate a greater sense of collectivism (9 reverse scores are included). One can define a group referred to in the scale in various ways. Examples of the questions include ‘I sacrifice self-interest for my group’ and ‘I act as fellow group members would prefer’.

#### Psychometric measures of general intelligence

RAPM, which is a widely used measure of general intelligence^36^, was utilised in the present study. This measure was adjusted to examine the effects of general intelligence on brain structures. For more details, please refer to our previous study^20^.

#### Behavioural data analyses

All behavioural data were analysed using IBM SPSS Statistics 22.0 software package (IBM Corp.; Armonk, NY, USA). Differences between males and females in terms of age and scores on the cognitive measures (RAPM and the collectivism score) were analysed using one-way ANOVA; a two-tailed *P* value < 0.05 was considered to indicate statistical significance.

### Image Acquisition

#### Structural MRI

All MRI data were acquired using a 3-T Philips Achieva scanner (Philips Medical Systems, Best, Netherlands). Three-dimensional high-resolution T1-weighted images were collected using a magnetisation-prepared rapid gradient-echo sequence with the following parameters: 240 × 240 matrix, TR = 6.5 ms, TE = 3 ms, TI = 711 ms, FOV = 24 cm, 162 slices, in-plane resolution = 1.0 × 1.0 mm, slice thickness = 1.0 mm, and scan duration = 483 s.

Diffusion-weighted data were acquired using a spin-echo echo-planar imaging (EPI) sequence with the following parameters: TR = 10293 ms, TE = 55 ms, FOV = 22.4 cm, 2 × 2 × 2 mm^3^ voxels, 60 slices, SENSE reduction factor = 2, and number of acquisitions = 1. The diffusion weighting was isotropically distributed along 32 directions (*b* value = 1,000 s/mm^2^), and three images with no diffusion weighting (*b* value = 0 s/mm^2^; b = 0 images) were acquired using the spin-echo EPI sequence (TR = 10293 ms, TE = 55 ms, FOV = 22.4 cm, 2 × 2 × 2 mm^3^ voxels, 60 slices). Acquisitions for phase correction and signal stabilisation were performed, but these data were not used as part of the reconstructed images. For more details regarding these procedures, please see our previous studies^20,30,37^.

### Pre-processing and Analyses of Structural Data

#### VBM data

All pre-processing of the T1 weighted image data was performed using Statistical Parametric Mapping software (SPM8; Wellcome Department of Cognitive Neurology, London, UK) according to the protocol described for voxel-based morphometry analyses in our previous study^18^. Using the new segmentation algorithm implemented in SPM8, T1-weighted structural images of each individual were segmented and normalised to the Montreal Neurological Institute space to yield images with 1.5 × 1.5 × 1.5 mm^3^ voxels using diffeomorphic anatomical registration through exponentiated lie algebra (DARTEL) registration process implemented in SPM8. In addition, we performed a volume change correction (modulation)^38^. All images were smoothed by convolving them using an isotropic Gaussian kernel of 6 mm full-width at half maximum (FWHM). These methods have been adapted from a previous study^18^. For additional details, please refer to our previous study^39^.

#### MD and FA data

All pre-processing and analyses of the imaging data were performed using SPM8 implemented in Matlab (Mathworks Inc.; Natick, MA, USA). The FA and MD maps were calculated from the collected images using a commercially available diffusion tensor analysis package (Philips Medical Systems, Best, Netherlands) on the MR console. These procedures involved corrections for motion and distortion caused by eddy currents, and all calculations were performed using a previously described method^40^. Briefly, the MD and FA images of the participants were normalised using a previously validated DARTEL-based registration process to develop images with 1.5 × 1.5 × 1.5 mm^3^ voxels. Furthermore, tissues that were least likely to be grey or white matter were carefully removed, and the images were smoothed by convolving them using an isotropic Gaussian kernel of 6-mm FWHM and normalised FA images were masked by a custom mask image indicating features highly likely to be white matter and then smoothed by a Gaussian Kernel of 6-mm FWHM. These methods have been adapted from a previous study^14^. For additional details, please see our previous studies^30,31^.

### Statistical Group-level Analyses of Imaging Data

Correction for multiple comparisons was performed using TFCE^41^ with randomised (5,000 permutations) nonparametric testing in the TFCE toolbox. A FWE-corrected threshold of *P* < 0.05 was applied. These methods have been adapted from a previous study^14^.

#### VBM data

A whole-brain multiple regression analysis performed in SPM8 was used to assess the association between rGMV (or rWMV) and collectivism scores. The covariates included sex, age, RAPM score, and TIV. For each covariate, the overall mean was used for mean centering. Analyses for rGMV and rWMV were performed in voxels for all subjects that showed a signal intensity of >0.05.

Subsequently, based on *a priori* hypothesis, we used ROI approaches to determine whether the rGMV correlates of the PFC areas included the Brodmann area 8, 9, 10, 11, 44, 45, 46, and 47. The ROI was constructed using the Brodmann area option of the WFU_PickAtlas tool (http://www.fmri.wfubmc.edu/cms/software#PickAtlas)^42,43^.

Sex differences in the correlates of collectivism were not the main purpose of the study; however, we also investigated whether the relationship between rGMV or rWMV and collectivism scores differed between males and females in a whole-brain analysis. We used a voxel-wise ANCOVA in which sex difference was a group factor using *t*-contrasts (using the full factorial option of SPM8). In this analysis, age, RAPM, TIV, and collectivism scores were modelled to enable unique relationships with rGMV (using the interaction option in SPM8 for each sex). TIV was modelled to have a common relationship with rGMV and rWMV across sexes.

#### MD and FA data

A voxel-by-voxel regression analysis was performed in SPM8 using the MD or FA value at each voxel as the dependent variable and age, sex, RAPM score, and collectivism score as the independent variables. The analysis using MD was limited to areas within the grey and white matter masks that were created using the procedures, whereas the analysis using FA was limited to areas within the white matter. We also investigated whether the relationship between MD or FA and collectivism scores differed between men and women by using the same method, rGMV or rWMV.

## Supplementary information

Supplementary Table 1

## Data Availability

The datasets generated during and/or analysed during the current study are available from the corresponding author on reasonable request.
