# Supplementary material for: Mean diffusivity related to collectivism among university students in Japan
Source: Sci Rep. 2019 Feb 4;9:1338. doi: 10.1038/s41598-018-37995-5 (PMC6362187; doi:10.1038/s41598-018-37995-5)
Supplement: Supplementary file 1 — Supplementary Table 1 [file 41598_2018_37995_MOESM1_ESM.doc]

**Mean diffusivity related to collectivism among university students in Japan**

Seishu Nakagawa^1,2*^, Hikaru Takeuchi^3^, Yasuyuki Taki^3,4,5^, Rui Nouchi^6,7^, Yuka Kotozaki^7^, Takamitsu Shinada^2^, Tsukasa Maruyama^2^, Atsushi Sekiguchi^4 ,8^, Kunio Iizuka^9^, Ryoichi Yokoyama^10^, Yuki Yamamoto^2^, Sugiko Hanawa^2^, Tsuyoshi Araki^11^, Carlos Makoto Miyauchi^2,12^, Daniele Magistro^13^, Kohei Sakaki^14^, Hyeonjeong Jeong^2,15^, Yukako Sasaki^14^, Ryuta Kawashima^7,14^

Institutions:

*1 Division of Psychiatry, Tohoku Medical and Pharmaceutical University, Sendai, Japan*

*2 Department of Human Brain Science, Institute of Development, Ageing and*

*Cancer, Tohoku University, Sendai, Japan*

*3 Division of Developmental Cognitive Neuroscience, Institute of Development, Ageing and Cancer, Tohoku University, Sendai, Japan*

*4 Division of Medical Neuroimaging Analysis, Department of Community Medical*

*Supports, Tohoku Medical Megabank Organization, Tohoku University, Sendai, Japan*

*5 Department of Nuclear Medicine and Radiology, Institute of Development, Ageing and Cancer, Tohoku University, Sendai, Japan*

*6 Creative Interdisciplinary Research Division, Frontier Research Institute for Interdisciplinary Science (FRIS), Tohoku University, Sendai, Japan*

*7 Smart Ageing International Research Center, Institute of Development, Ageing and Cancer, Tohoku University, Sendai, Japan*

*8 Department of Psychosomatic Research, National Institute of Mental Health, National Center of Neurology and Psychiatry, Kodaira, Tokyo, Japan.*

*9 Department of Psychiatry, Tohoku University Graduate School of Medicine, Sendai, Japan*

*10 School of Medicine, Kobe University, Kobe, Japan*

*11 ADVANTAGE Risk Management Co., Ltd, Tokyo, Japan*

*12 Department of Language Sciences, Graduate School of Humanities, Tokyo Metropolitan University, Tokyo*

*13 Department of Sport Science, School of Science and Technology, Nottingham Trent University*

*14 Advanced Brain Science, Institute of Development, Aging and Cancer, Tohoku University, Sendai, Japan*

*15 Graduate School of International Cultural Studies, Tohoku University, Sendai, Japan*

Supplemental Table 1**.**

**Brain regions exhibiting a correlation between rGMV (or rWMV) and collectivism scores (*P* < 0.001 uncorrected (*t* = 3.5) at the whole-brain level).**

| Brain region | G/W | R/L | x | y | z | *t-*value | Cluster size (k_E_) |
| --- | --- | --- | --- | --- | --- | --- | --- |
| Supra marginal gyrus | G | R | 59 | -35 | 39 | 4.31 | 269 |
| Precuneus | G | L | -14 | -62 | 42 | 3.95 | 68 |
| Precentral gyrus | G | R | 53 | -12 | 41 | 3.83 | 158 |
| Superior parietal gyrus | G | R | 17 | -56 | 72 | 3.78 | 40 |
| Superior parietal gyrus | G | L | -38 | -54 | 63 | 3.58 | 61 |
| Postcentral gyrus | W | R | 56 | -20 | 33 | 3.56 | 44 |
| Precuneus | W | L | -12 | -72 | 36 | 3.51 | 14 |

G, gray matter; W, white matter; L, left; R, right.
